# Report of the EORTC-PAMM Meeting, Brussels, 16–18 March 2009: new strategies for a targeted and personalised therapy of cancer

**DOI:** 10.3332/ecancer.2009.141

**Published:** 2009-04-02

**Authors:** S Camporesi

**Affiliations:** European School of Molecular Medicine, Via Adamello, 16-20139 Milan, Italy

## Introduction

For the first time, the 2009 Pharmacology and Molecular Mechanisms (PAMM) winter meeting was organized in connection with the EGAM (EORTC Groups Annual Meeting) in Brussels. Furthermore, the PAMM participated in an additional plenary session together with the PathoBiology Group and the functional imaging group during the EGAM itself, in which the Laboratory Research Division summarized their expertise in translational research and gave an overview of what it can offer to the Disease Oriented Groups. The emphasis of the joint meeting was on novel concepts in cancer treatment and on new approaches to monitor therapy and implement personalized strategies.

Three main subtopics could be possibly canvassed out of whole meeting, namely, (a) apoptosis and signalling, (b) cancer stem cells, (c) cancer genome and epigenome profiling.

### (a) Targeting apoptosis

Most anti-cancer therapies act by triggering apoptosis in cancer cells. Therefore, defects in apoptosis programmes, for example aberrant expression of anti-apoptotic proteins, may render cancer cells resistant to treatment. Indeed, deregulation of apoptosis is one of the hallmarks of malignant cells, allowing their survival in the context of an altered genome and in harsh tumour environments with low levels of oxygen and nutrients. Understanding of the molecular mechanisms underlying apoptosis suppression in cancer has provided rationales for the design of apoptosis targeted therapies.

One strategy was illustrated by Simone Fulda (University Children’s Hospital, Ulm, Germany) and involves the targeting of inhibitors of apoptosis proteins (IAPs), which are expressed at high levels in many human cancers and block apoptosis by inhibiting effector caspases. Fulda showed how targeting XIAP (one member of the IAP family) by RNA interference-mediated knockdown or small molecule inhibitors cooperates with TRAIL (tumour-necrosis-related apoptosis-induced ligand) to suppress growth in both ***in vitro*** and ***in vivo*** models of pancreatic cancer and in childhood acute leukaemia cells [14,25]. In this context, IAP inhibitors appear to be promising therapeutic tools, and they have recently entered early clinical trials in the form of small peptides.

Nadia Zaffaroni (Fondazione IRCCS Istituto Nazionale dei tumouri, Milan, Italy) showed promising ***in vitro*** and ***in vivo*** data on ‘survivin’ inhibition, another member of the IAP family of apoptosis inhibitors. Survivin is a bifunctional protein that in addition to acting as a suppressor of programmed cell death also plays a central role in cell division. Owing to its massive up-regulation in human tumours and its involvement in cancer progression and treatment resistance, survivin is currently undergoing extensive investigation as a promising target for new anti-cancer interventions. Zaffaroni showed how the down-regulation of this protein, accomplished by means of various strategies (including the use of antisense oligonucleotides, small interfering RNAs, ribozymes, dominant negative mutants and small molecules antagonists) reduced tumour growth potential, increased the apoptotic rate and sensitized tumour cells to chemo- and radiotherapeutic agents in different tumour pre-clinical models [18]. The first survivin inhibitors have already reached the clinic: the YM155 molecule is currently in phase 1–2 studies [22]. However, due to its central role in promoting cell division in normal cells, the effects of survivin disruption on normal tissues and the related possible toxicities must be further investigated.

Frank A Kruyt (University Medical Center Groningen, the Netherlands) presented his work on bortezomib, an interesting compound that can reversibly inhibit the proteasome and induce mitochondrial-dependent apoptosis [26]. Kruyt argued that bortezomib may be an interesting targeting moiety for non-small-cell lung cancer (NSCLC), a disease with poor survival rates, which represents 80–85% of lung cancer cases. The combined administration of TRAIL/bortezomib proved highly effective in inducing mitochondrial-dependent apoptosis in both ***in vitro*** and ***in vivo*** models of NSCLC. Mechanistic studies are ongoing to better understand the favourable drug interactions. In the clinic, recombinant TRAIL preparations and agonistic antibodies are under examination and combination strategies are in progress, including mapatumumab (TRAIL-R1/DR4 mAb) and bortezomib in multiple myeloma [6–8].

### (b) Targeting cancer stem cells

The idea of targeting tumour stem cells for cancer treatment first became prominent in the 1970s with the introduction of the ‘human tumour stem cell assay’ by Hamburger and Salmon [15]. Twenty years after that first report, interest in the tumour stem cell has re-emerged out of the increasing understanding of normal human stem cell biology and work documenting the existence of human leukaemic stem cells bearing characteristic cell surface markers [1]. Interest in targeting tumour stem cells has been based on the notion that these cells resist currently available therapies and contribute to the regrowth of tumours following chemotherapy. Robert H Shoemaker (Division of Cancer Treatment and Diagnosis, National Cancer Institute, USA) presented data on the characterization of the *in vitro* drug sensitivity phenotype of isolated tumour stem cells from glioma samples and established tumour cell lines. A high degree of heterogeneity in the expression of several markers tested and the existence of sub-populations of tumour stem cells expressing different sets of markers were pointed out, together with a high heterogeneity in the resistance to more than 50 drugs tested [20]. Some unresolved issues in the quantitation of the fraction of tumour stem cells in patient samples were also highlighted, namely the need to standardize the experiments, and the results, according to the strain of immunodeficient mice used, the site and mode of implantation.

Louis Vermeulen (Academic Medical Center, Amsterdam, the Netherlands) focused on colon cancer stem cells. Colon carcinomas often consist of a range of cells expressing various differentiation markers. In analogy to normal colon crypts, goblet-like, enterocyte-like or enteroendocrine-like differentiation patterns are observed. Vermeulen argued in favour of this set of separate differentiation routes emanating from a single-colon cancer stem cells. To support his claim, he presented data showing how a primary tumour can be regenerated in a mouse using single-cell cloned cancer stem cells (CSCs), either after *in vitro* culture or directly from the human tumour specimen [24]. In addition, Vermeulen showed recent work, revealing that regulation of the Wnt signalling cascade in colorectal cancer cells harbouring APC mutations occurs and is related to the CSC phenotype both *in vitro* and *in vivo*. Currently, his group is investigating the molecules involved in the regulation of the aberrant Wnt cascade, which may serve as a promising future therapeutic target.

The relationship between cancer stem cells and circulating cancer cells, and their potential for metastasis, was investigated in the talks given by Sieuwerts and Fodstad.

Anieta M Sieuwerts (Josephine Nefkens Institute, Erasmus MC, Rotterdam, the Netherlands) focused on the CellSearch™ method to identify circulating tumour cells (CTCs), and on its pitfall in detecting the aggressive subtype of breast CTCs responsible for metastasis and relapse, namely ‘normal-like breast tumour cells’. Indeed, the CellSearch™ isolation method, which uses epithelial cell adhesion molecules such as EpCAM to detect such tumour cells, is not able to recognize normal-like breast cancer cells, which, in general, have aggressive features and are negative for the expression of EpCAM. Therefore, new tests that identify antibodies, which specifically recognize normal-like breast tumour cells must be pursued. Sieuwerts proposed useful criteria for the selection of addition cell surface antigens [21]. Furthermore, epithelial mesenchymal transition (EMT) is a process that has been linked to the ability of breast cancer stem cells to give rise to metastasis [19,17]. Sieuwerts pointed out how the normal-like breast cancer subtype is the main subtype with EMT characteristics. Therefore, these cells are important targets for the development of individualized therapies. According to Sieuwerts, these cells may represent a subset of the cancer stem cells present in the original tumour lesion.

The relationship between CSCs and CTCs has been investigated also by Øystein Fodstad (Institute for Cancer Research, Norwegian Radium Hospital, Oslo, Norway), who focused on methods for detecting disseminated tumour cells. The presence of micro-metastatic tumour cells in peripheral blood and bone marrow has been shown to correlate with poor outcome. The technologies used to detect micro-metastatic cells have several limitations in terms of specificity and sensitivity and of false positives and false negatives, as the huge discrepancy in the fraction of breast cancer stem cells in published results demonstrates. In general, methods must cope with two questions. First, how do we know we are actually detecting tumour cells? As for the second, Fodstad raised a point discussed also by Sieuwerts: do micro-metastatic cells express stem cell markers and what is their relationship with cancer stem cells? Fodstad illustrated the work of his group on the development of an immunomagnetic method for selective isolation of micro-metastatic cells in body fluids and solid tissues, simultaneously examining the expression of cell membrane markers by the use of non-magnetic fluorescent micro-particles coated with relevant antibodies. These live, bead-bound cells are then collected and used for RT-PCR and array comparative genomic hybridization (aCGH) studies and for the comparison of cells from different compartments [11].

### (c) Profiling the cancer genome and epigenome

A novel approach exploiting this last tool (aCGH studies) for diagnosis and patient treatment was illustrated by Bauke Ylstra (VU University Medical Center, Amsterdam, the Netherlands). This method is based on the assumption that patient-tailored medicine requires matching of the most effective therapy with the molecular characteristics of a cancer. Therefore, the molecular heterogeneity of the individual patient’s tumour needs to be recognized. Chromosomal copy number aberrations (CNAs) offer opportunities to stratify patient samples for therapy [5] and aCGH is the high-resolution laboratory technique of choice to detect such CNAs with high resolution and on a genome wide scale [28]. Unsupervised clustering of chromosomal copy number profiles allows sub-classes of mammary tumours, which are tightly linked to survival in independent datasets to be distinguished [2,3]. In addition, Ylstra pointed out how CNAs could function as markers to predictor response to chemotherapy in advanced colorectal cancer. aCGH can also be used for identification of novel drug targets targeting small, focal aberrations less than 1 Mb long. In total, Ylstra showed how CNAs can serve as a marker for better cancer classification, prognosis, and outcome prediction. He also argued in favour of aCGH as a better diagnostic and prognostic tool compared to expression profiling, since DNA is a more stable molecule than RNA. The value of aCGH clustering can however be increased, Ylstra contended, by coupling CNAs profiles with RNA profiles.

John Martens (Josephine Nefkens Institute, Rotterdam, the Netherlands) focused instead on the other side of the ‘genomic tool-coin’ for the molecular understanding of cancer and for guiding personalized therapy. He argued in favour of micro-RNAs as prognostic and predictive tumour markers, in particular in breast cancer [13]. Micro-RNAs (miRNAs) are small ribonucleotides (16–29 nt) that use the endogenous RNA interference pathway to modulate gene expression in a tissue and developmental stage-dependent manner. Martens and co-authors presented an innovative study in breast cancer aimed at assessing which miRNAs are associated with more aggressive tumour phenotype and poor prognosis or clinical benefit from endocrine therapy. An analysis of 249 miRNAs in 38 primary breast cancers identified four miRNAs correlated with poor prognosis and three others with a favourable response to endocrine therapy with tamoxifen. Then, using the available gene expression data from these cohorts of patients Martens and co-authors determined which biological pathways were co-expressed with the identified prognostic or predictive microRNAs. To conclude, Martens stressed how global miRNA signatures can lead to the identification of miRNAs predictive of prognosis, response and to novel targeted therapies [10].

The most original talk was undoubtedly given by Terry Jones (Manchester, England), the only speaker who focused on the innovative role of molecular imaging in clinical trials. In particular, Jones and co-authors talked about positron emission tomography (PET) as a tool for early clinical trials and for filling in the gap between the laboratory and the clinic [27]. Indeed, as a means for supporting drug development, PET offers measurements of regional tissue pharmacokinetics and pharmacodynamics and provides quantitative ‘proof-of-concept’ information on mechanisms and efficacies of action. According to Jones, the future of cancer clinical trials is in imaging-based microdose studies, such as phase 0 studies, which would offer opportunities for rapid first into humans trials and avoid the need of massive toxicology testing [9]. However, the field of molecular imaging studies is still underdeveloped, and Jones pointed out the challenges that are slowing down the potential use of PET to support phase 1 oncology studies, such as the need to discover specific-imaging biomarkers for tumour tissue.

## Conclusion

The first PAMM-EORTC joint meeting pointed out some common challenges in the direction towards targeted, personalized therapy for cancer. One of these promising routes is through targeting cancer stem cells.

The cancer stem cell hypothesis proposes that cancer stem cells are a minority population of self-renewing cancer cells that fuel tumour growth and remain in patients after conventional therapy has been completed. The model predicts that effective tumour eradication will require obtaining agents that can target cancer stem cells whilst sparing normal stem cells. Vermeulen hinted at the problem of the integration of this model within the model of carcinogenesis postulated by Nowell and Vogelstein, which describes the formation of a tumour by the sequential accumulation of mutations in oncogenes and tumour suppressor genes [23]. Vermeulen also dwelled on the problem of the definition of these cells, since and accurate and common definition is critical to enable researchers working in the same or different systems to compare cells exhibiting a common set of properties. The term ‘cancer stem cell’ has led to some confusion, and some scholars prefer to use the term ‘tumour initiating cell’ [16].

According to Vermeulen, the consensus definition of a cancer stem cell that was reached the AACR 2006 Workshop (‘a cell within a tumour that possess the capacity to self-renew and to cause the heterogeneous lineages of cancer cells that comprise the tumour’) is a satisfactory solution, even though it is only an operational definition [4].

Shoemaker pointed out some of the challenges that may hinder the translation of the cancer stem cell promise to the clinic. Indeed, the moving target nature of cancer stem cells may present a serious obstacle for drug development. To achieve effective implementation of new therapies, physicians will require methods of determining the type (or types) of cancer stem cells present in a given patient’s tumour. It is therefore of pivotal importance that agents directed against cancer stem cells discriminate between tumour stem cells and normal stem cells.

The problem of the discrimination of the normal from the pathological compartment (and the related point of cancer drug toxicity) is not limited to compounds targeting cancer stem cells; Zaffaroni also highlighted this issue describing what needs to be done in targeting apoptosis, such as when inhibiting survivin.

The still incomplete clarification of the relationship between cancer stem cells and circulating tumour cells is at present another hurdle for the translation of the model to the clinic. Both the presentations by Sieuwerts and Fodstad elucidated different aspects of this problem, which surely has significant therapeutic implications. Indeed, some questions still remain open: Are circulating tumour cells a subset of cancer stem cells? What is the role of the epithelial mesenchymal transition in the acquisition of an aggressive phenotype and of the metastatic potential? Are micro-metastatic cells the same thing as circulating tumour cells?

Genomic tools for cancer profiling, such as the ones illustrated by Ylstra and Martens, might be the solution for this kind of question, as they may help identifying DNA and RNA signatures predictive of clinical response and outcome. The ultimate goal is the stratification of patients according to their tumour signatures and the development of a more effective personalized treatment. To achieve these results, advancement in molecular imaging tools, such as PET-based clinical trials, need to be boosted, as underlined by Terry Jones. The potentiality of the molecular imaging studies is still not fully developed, due to the lack of suitable biomarkers and to the sub-optimal receptivity of the field to the existing guidelines on microdose studies, one of the main applications of the new phase 0 trials [12].

To conclude, several roads towards the shared goal of targeted and personalized cancer therapy have been paved. These go through the modulation of apoptosis pathways, cancer stem cells and micro-metastic cells, chromosomal aberrations and tumour-upregulated miRNAs and the stratification of patients according to their tumour DNA and RNA profiles. Different routes need not be necessarily mutually exclusive, since they could greatly profit by favourable interactions between diverse expertises. One example of this ‘virtuous circle’ was demonstrated by the fruitful exchanges of ideas between Laboratory Research Division and Disease Oriented Groups that took place this year in Brussels. Let us hope it is not the first and last time there is a joint PAMM-EORTC meeting!
